# Age-related hearing loss persisted in mice that had demonstrated improved healthspan, reduced inflammaging and extended lifespan following expression of the naked mole-rat transgene for hyaluronan synthase 2

**DOI:** 10.3389/fragi.2026.1936677

**Published:** 2026-08-27

**Authors:** Connor E. Owens, Aiden J. Borruso, Kevin A. Place, Andrei Seluanov, Vera Gorbunova, Patricia M. White, Ned J. Place

**Affiliations:** 1 Department of Population Medicine and Diagnostic Sciences, Cornell University, College of Veterinary Medicine, Ithaca, NY, United States; 2 Department of Biology, University of Rochester, Rochester, NY, United States; 3 Department of Medicine, University of Rochester Medical Center, Rochester, NY, United States; 4 Department of Neuroscience, University of Rochester School of Medicine and Dentistry, Rochester, NY, United States

**Keywords:** age-related hearing loss, auditory brain stem response, hyaluronan, naked mole-rat, presbycusis

## Abstract

**Background:**

Approximately one in three adults aged 65–74 years reports hearing difficulty, including age-related hearing loss (AHL, presbycusis). The C57BL/6 mouse is widely used in preclinical AHL research because it develops AHL relatively early. Calorie restriction (CR) attenuates AHL and improves healthspan and longevity in this strain. A recently developed transgenic C57BL/6 mouse expressing naked mole-rat hyaluronan synthase 2 (*nmrHas2*), which produces very high molecular mass hyaluronan (vHMM-HA), also exhibits improved healthspan, reduced inflammaging, and increased longevity. Therefore, we evaluated *nmrHas2* mice for evidence of alleviated AHL.

**Objective:**

To determine whether AHL is attenuated in *nmrHas2 mice*.

**Methods:**

This study supplemented a larger investigation of *nmrHas2* and female reproductive aging. Tamoxifen administered at 1 month induced ubiquitous *nmrHas2* expression in *nmrHas2+* females; *nmrHas2−* controls received tamoxifen or vehicle. Auditory brainstem response (ABR) testing was performed approximately 2 weeks before mice reached 3 or 12 months of age. Cochleae and reproductive tissues were collected following euthanasia approximately 2 weeks later.

**Results:**

ABR thresholds to clicks and six tones (4–32 kHz) showed marked hearing loss from 3 to 12 months of age in both *nmrHas2+* mice and controls, with no evidence that *nmrHas2* attenuated AHL. Cochlear *nmrHas2* expression in *nmrHas2+* mice was confirmed by quantitative RT-PCR.

**Conclusion:**

Ubiquitous *nmrHas2* expression did not attenuate AHL in C57BL/6 mice despite previously reported benefits for other aging phenotypes. These findings contrast with CR, suggesting that vHMM-HA and CR influence aging through distinct mechanisms.

## 1 Introduction

Age-related hearing loss (AHL, also called presbycusis) is typically caused by irreversible damage to cochleae that occurs during aging (Lin et al., 2011; Yang et al., 2015). In humans, approximately one in three individuals 65–74 years of age self-reports difficulty in hearing, including AHL (Cassarly et al., 2020). Understanding potential mechanisms that contribute to or alleviate AHL is key to the development of successful therapeutics that attenuate AHL progression. This is increasingly important as global longevity increases, with average life expectancy increasing from 66.8 years in 2000 to 73.6 years in 2019 (Wang et al., 2020). Interestingly, pre-clinical studies in mice have shown that calorie restriction (CR) essentially prevents AHL when tested at 12 months of age (Someya et al., 2007; 2010). In addition to alleviating AHL, CR also improves the healthspan of mice, reduces inflammaging, and increases longevity (Kökten et al., 2021). These are traits that CR mice share with a recently developed mouse model that expresses an inducible transgene for naked mole-rat (NMR) hyaluronan synthase 2 (*nmrHas2*) (Zhang et al., 2023). As such, our objective for this study was to test the hypothesis that AHL would be reduced following induction of *nmrHas2* expression at 1 month of age, followed by continuous, ubiquitous *nmrHas2* expression thereafter (Zhang et al., 2023).

Naked mole-rat *Has2* synthesizes a form of hyaluronan (HA, hyaluronic acid) with a very high molecular mass (vHMM-HA) that has been associated with the NMR’s resistance to cancer (Tian et al., 2013), and its extreme longevity (40+ years, Ruby et al., 2018). These attributes have been credited, at least in part, to the superior cytoprotective properties of the NMR’s vHMM-HA (Takasugi et al., 2020). Hyaluronan is an unbranched disaccharide glucuronic acid/N-acetylglucosamine polymer and a major component of the extracellular matrix. The NMR vHMM-HA (6–12 MDa) is five to six times larger than mouse or human HMM-HA (0.5–3 MDa and 0.5–2 MDa, respectively) (Tian et al., 2013). In mice and humans, HMM-HA undergoes degradation and fragmentation, and when fragments are not properly cleared, this leads to the accumulation of low molecular mass (LMM) HA, which is biologically active and can induce inflammation and fibrosis (Robert et al., 2010; Papakonstantinou et al., 2012; Monslow et al., 2015). The vHMM-HA in NMRs is resistant to this degradation (Sato et al., 2024). Additionally, binding of vHMM-HA to its primary receptor, CD44, induces expression of cytoprotective pathways and prevents binding of LMM-HA to CD44 (Takasugi et al., 2020).

The *nmrHas2* mouse is the subject of an ongoing project that aims to determine whether female reproductive aging is attenuated at 12 months of age after induction of the *nmrHas2* transgene at 1 month of age. In addition to their exceptional longevity, female NMRs are remarkable for the lack of any age-associated decline in fertility (Buffenstein, 2008). As a supplement to the study on female reproductive aging, we added an investigation of AHL, which is also evident by 12 months of age in the background strain for the *nmrHas2* mouse (C57BL/6). This is the same mouse strain for which CR was found to alleviate AHL, and at the same age when we interrogated female *nmrHas2* mice for signs of reduced reproductive aging. By evaluating auditory and reproductive outcomes in the same mice to determine whether CR mice and *nmrHas2* mice share not only improved health- and life-spans, but also two additional phenotypes that have been previously reported in CR mice–alleviated AHL (Someya et al., 2007; 2010) and delayed female reproductive aging (Prosczek et al., 2025). The results of the auditory brainstem response (ABR) testing of *nmrHas2* mice and controls are reported herein, while the comprehensive assessments of reproductive aging in these mice will be reported elsewhere.

## 2 Materials and methods

### 2.1 Animal husbandry

Female mice were group housed and provided with standard chow diet and water *ad libitum*. All mice were housed under controlled conditions of temperature (range 21 °C–24 °C), 12:12 h light:dark cycles, and relative humidity of 40%–50%. All experimental procedures were approved by Cornell University’s Institutional Animal Care and Use Committee (protocol number 2020-0057).

### 2.2 Transgenic animal design and use

**FIGURE 1:**
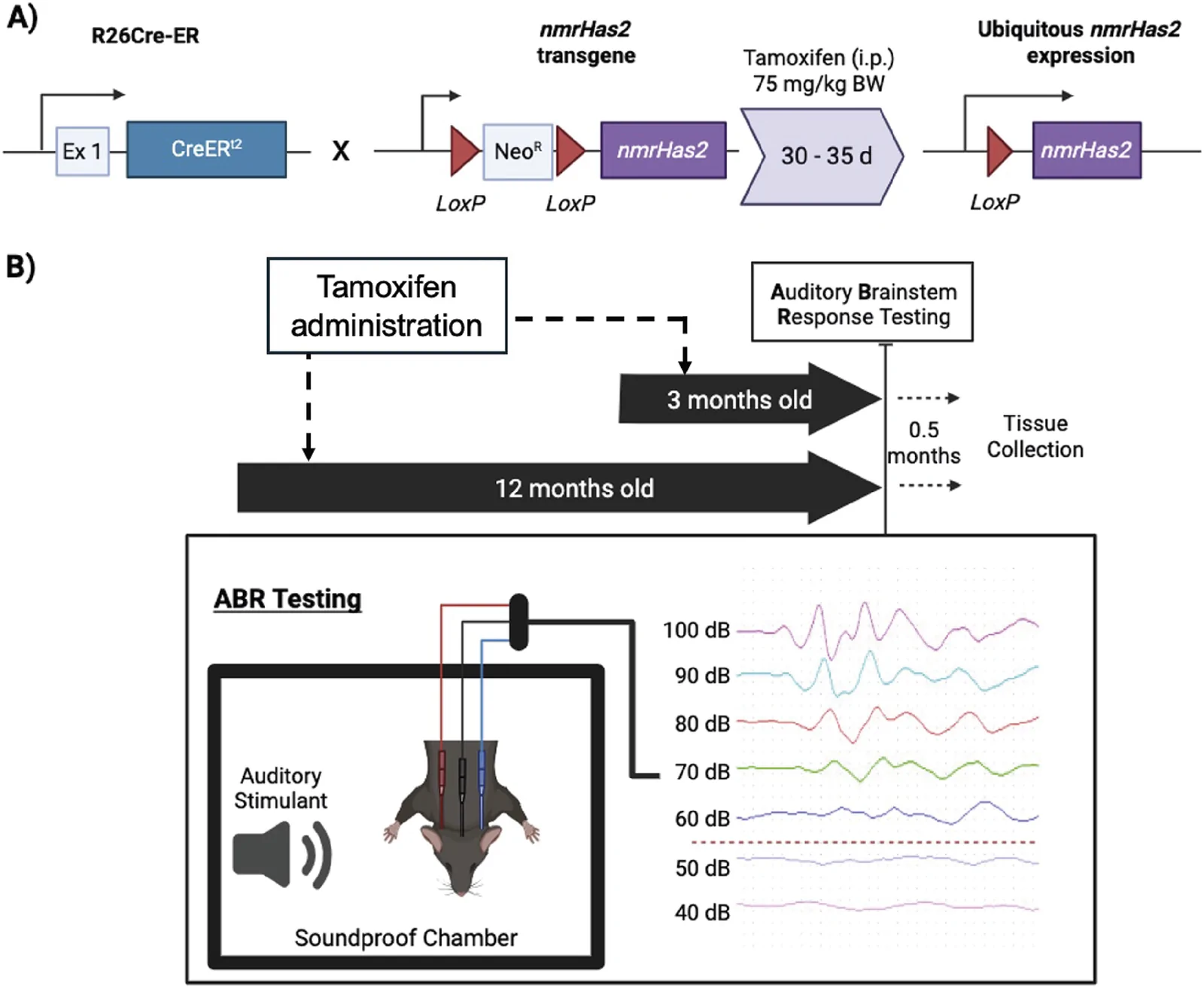
Outline of transgenic mouse model and experimental design. **(A)** Experimental mice were generated by cross breeding sires that are homozygous for the CreER^t2^ recombinase in exon 1 of *Rosa26* (Cre-ER^t2^) with dams that are heterozygous for the *nmrHas2* transgene, for which the upstream Neo^R^-LoxP cassette prevents expression. Litters produced contained *nmrHas2*+/− x CreER^t2+/−^ (nmrHas2+) and *nmrHas2−/−* x CreER^t2+/−^ (*nmrHas2-*) offspring. One-month-old female offspring were given daily intraperitoneal injections of 75 mg/kg BW of tamoxifen for 5 days to induce *nmrHas2* expression in nmrHas2+ mice and equivalent volumes of corn oil (Vehicle) to control for potential effects of tamoxifen. **(B)** Mice were raised to 3 months or 12 months of age and underwent auditory brainstem response (ABR) threshold testing. The response threshold was determined as the lowest stimulus level (in decibels, dB) that elicited a wave-like pattern of response (in the example, it would be classified as 60 dB).

We generated transgenic mice as previously described (Zhang et al., 2023) (Figure 1A). Briefly, we cross bred *nmrHas2* (C57BL/6NCrl, Charles River Lab, code 027) and homozygous *R26*-*creER* ^*t2*^ (B6129SF2/J, JAX, #101045) to generate heterozygous *nmrHas2* dams and homozygous *R26-creER* ^*t2*^ sires. At 21 days of age, we weaned female offspring, placed an ear tag in one pinna, and collected tissue from the opposite pinna for genotype determination. These females were primarily interrogated for signs of ovarian aging and reduced fertility and fecundity at 12 months of age; however, a subset of animals underwent ABR testing to assess auditory function approximately 2 weeks prior to reaching their target ages for the collection of reproductive tissues. We initiated ABR testing after some females, particularly 3-month-olds, had already been euthanized for the collection of reproductive tissues, which led to an unbalanced design in terms of sample sizes. However, our sample size analyses determined four animals per group would detect differences in ABR thresholds of ≥ 20 dB between young (3 months) and old (12 months) animals at α = 0.05 and β = 0.80. We based these calculations and assumptions on the 35–50 dB differences in ABR thresholds between 2- and 12-month-old mice as reported by Someya et al. (2010). And for the primary contrast between 12-month-old *nmrHas2+* and *nmrHas2-*mice previously treated with tamoxifen, we determined a sample size of eight animals per group could detect differences in ABR thresholds of ≥ 10 dB at α = 0.05 and β = 0.80, again based on results reported by Someya et al. (2010).

The experimental design is outlined in Figure 1B. At 30 days of age, we administered 75 mg/kg tamoxifen (Tam; Sigma-Aldrich, St. Louis, MO) in sterile corn oil or an equal volume of corn oil alone (Veh; Sigma-Aldrich, St. Louis, MO) to mice via intraperitoneal (i.p.) injection daily for 5 days. For the tamoxifen-treated *nmrHas2+* mice, this induced ubiquitous, life-time expression of *nmrHas2*. We also administered equivalent volumes of corn oil to a subset of *nmrHas2-* mice to control for the potential effects of Tam treatment. Due to unanticipated expression of *nmrHas2* in ovaries of *nmrHas2+* mice not administered tamoxifen, only *nmrHas2+* mice given tamoxifen were included in these experiments. The ages at which we collected reproductive organs from mice (3 and 12 months) corresponded with previous literature for normal hearing and AHL in C57BL/6 mice with the *Cdh23* SNP (Nemoto et al., 2004; Noben-Trauth et al., 2003; Yang et al., 2015). Animals fell into six groups based on genotype, age, and treatment combination: *nmrHas2-* 3 m Tam (*n* = 4); *nmrHas2-* 3 m Veh (*n* = 6); *nmrHas2-* 12 m Tam (*n* = 9); *nmrHas2-* 12 m Veh (*n* = 8); *nmrHas2+* 3 m Tam (*n* = 5); *nmrHas2+* 12 m Tam (*n* = 8).

### 2.3 Auditory brainstem response (ABR) measurement and analysis

Approximately 2 weeks before reaching the target ages for euthanasia and tissue collection, we performed ABR testing on female mice. Testing occurred between the hours of 9 AM and 12 PM EST, and prior to each day’s testing, we calibrated the instrument for clicks and pip tones at all frequencies. The trapezoid filter settings included a target level of 100 dB, maximum correction 20 dB, and filter length 512. We deeply anaesthetized mice using an intraperitoneal injection of ketamine (80 mg/kg animal weight) and xylazine (3 mg/kg animal weight). If after 10 min the desired plane of anesthesia had not been achieved (i.e., no response to foot pad pinch), an additional half dose of ketamine and xylazine was administered. We placed mice on a non-electric isothermal warming pad (Braintree Scientific, Inc., Braintree, MA) within a sound-proof chamber (Sound Room Solutions, Inc., Glen Cove, NY). We inserted three needle electrodes subcutaneously at the forehead and near each pinna. We placed the transducer 5 cm from the left pinna. Electrodes were connected by cables to the ABR testing system (Tucker-Davis Technologies, Inc., Alachua, FL). We first evoked ABR responses using a 50 μsecond click stimuli. Immediately thereafter, we evoked ABR responses using 1 millisecond pure tones at frequencies of 4, 8, 12, 16, 24, and 32 kHz, with evoked responses averaged across 512 sweeps. We recorded responses for each stimulus level in 10 dB steps from 100 dB to 10 dB sound pressure level (SPL) for both clicks and tones. Following the completion of the ABR testing and recovery from anesthesia, we returned mice to their pre-testing housing arrangement. Repeatability was not assessed, which is standard practice for ABR testing in mice. The ABR threshold was defined as the lowest SPL sufficient to elicit at least one peak that was consistent with an ABR waveform (Figure 1B). A single researcher (K.A.P.) who was unaware of the animal’s age, genotype, and Tam/Veh scored the ABR recordings for thresholds. Scoring ABR thresholds followed training sessions involving results from 10 randomly selected mice for which the scorer agreement between the trainee and trainer (N.J.P.) was 100%. For the purpose of statistical analyses, we recorded animals that did not elicit a peak at any stimuli as having an ABR threshold of 110 dB, which is one 10 dB step above the maximum stimulus tested. A single researcher who was unaware of each animal’s age, Tam vs Veh treatment, and genotype scored ABR thresholds for all click and tone tests.

### 2.4 Tissue collection, RNA extraction, and RT-qPCR

Mice reached target ages approximately 2 weeks after ABR testing, at which time we individually euthanized mice via CO_2_ inhalation at a flow rate of 3.5 L/min followed by at least 3 min without any respiratory movement. Thereafter, we removed the cochleae by microdissection and processed them for future interrogation if the ABR results indicated AHL had been attenuated or alleviated. We also collected cochleae from a subset of young mice (n = 3 per genotype) for RNA extraction to confirm *nmrHas2* expression in the cochlea. For each mouse, we placed both isolated cochleae in a sterile 12-well plate on ice with chilled lysis buffer from the Qiagen RNeasy Plus Kit (QIAGEN; Hilden, Germany). We then broke apart cochleae using sterile forceps and scraped out sensory areas to expose cells to the lysis buffer. We further minced tissue first using sterile scalpel blades followed by homogenization in lysis buffer using Bead-Ruptor Pre-Filled Metal Bead Tubes (Omni International; Kennesaw, GA, United States). We extracted RNA from the homogenized tissue using the Qiagen RNeasy Plus Kit (QIAGEN; Hilden, Germany) and QIAShredder Columns (QIAGEN; Hilden Germany) according to manufacturer’s instructions.

We performed qRT-PCR using PowerTrack™ SYBR Green Master Mix (Applied Biosystems; Waltham, MA, United States) to confirm cochlear expression of *nmrHas2* in nmrHas2+ mice. We used approximately 700 ng of extracted RNA to make cDNA using the SuperScript III First-Strand Synthesis kit (Invitrogen; Waltham, MA, United States). Due to the high similarity of the *Has2* sequences in NMR and mice, we designed primers that were preferential to the NMR *Has2* mRNA sequence over the mouse *Has2* sequence, but not entirely exclusive. We performed qRT-PCR using primers preferential for *nmrHas2* (Forward primer: GAG AGC TCA CAG GTG ACA CAG; Reverse primer: GGA GTC ACA AAC CTG CAC ATA ATC CAC A). We used previously generated cDNA libraries from NMR ovaries as a positive control for *nmrHas2* expression and primers specific for mouse *Has2* as a positive control for qRT-PCR in *nmrHas2-*mice. We performed qRT-PCR on an ABI 7500 FAST instrument (Applied Biosystems; Waltham, MA, United States)) for 40 cycles (95 °C × 5 m; 40 cycles of [95 °C × 15 s, 60 °C × 30 s, 72 °C × 60 s]; then 72 °C × 5 m, hold at 10 °C) with all samples and the no-template control run in duplicate and a Ct cut-off of 35 cycles. We calculated the relative *Has2* expression using the ^ΔΔ^Ct method and *Gapdh* as the reference/housekeeping gene (Forward primer: TCA CTG CCA CCC AGA; Reverse primer: GAG GGA CAC ATT GGG GGT AG), and the value for *nmrHas2-*mice set at 1. The ABI 7500 FAST does not perform a melt curve, but the single band on the gel electrophoresis and sequencing results of the excised band support the production of a single qRT-PCR product. We performed gel electrophoresis and gel extraction using the Qiagen QIAQuick Gel extraction kit (Qiagen; Hilden, Germany) to isolate qRT-PCR products and confirm their sequences. We performed Sanger sequencing of qRT-PCR products at the Cornell Bioinformatics Resource Center using Big Dye Terminator Cycle (Applied Biosystems; Waltham, MA) and confirmed the sequence similarity of qRT-PCR products to the NMR *Has2* sequence.

### 2.5 Statistical analysis

We performed all statical tests in JMP Pro, ver. 17.0.0 (SAS Institute Inc.; Cary, NC, United States). To assess whether ABR thresholds were different based on age, genotype, or tamoxifen treatment, we first performed a generalized linear model for clicks and all tones. The tone frequencies were modeled separately, and therefore, inclusion of ‘animal’ as a random effect was unnecessary. The ABR threshold was the outcome variable and fixed effects were genotype, age, tamoxifen treatment, and genotype × age interaction. We performed the following planned *post hoc* contrasts for clicks and all tones: 3 m and 12 m *nmrHas2-*, 3 m and 12 m *nmrHas2+*, and 12 m *nmrHas2-*and 12 m *nmrHas2+*. We did not observe an effect based on tamoxifen treatment, so this effect was not examined in *post hoc* contrasts. Whereas the assignment of 110 dB as the ABR threshold for instances when no ABR waveform was identified at any amplitude results in right censoring of the data, this does not affect outcome of the primary contrast between *nmrHas2+*/12 m/Tam and *nmrHas2-*/12m/Tam because if the value were >110 dB, it would not change the interpretation. Additionally, the proportions of 12-month-old animals that were assigned an ABR threshold of 110 dB were not statistically different in *nmrHas2* positive and negative mice at any frequency (data not shown).

## 3 Results

### 3.1 ABR testing results

**FIGURE 2:**
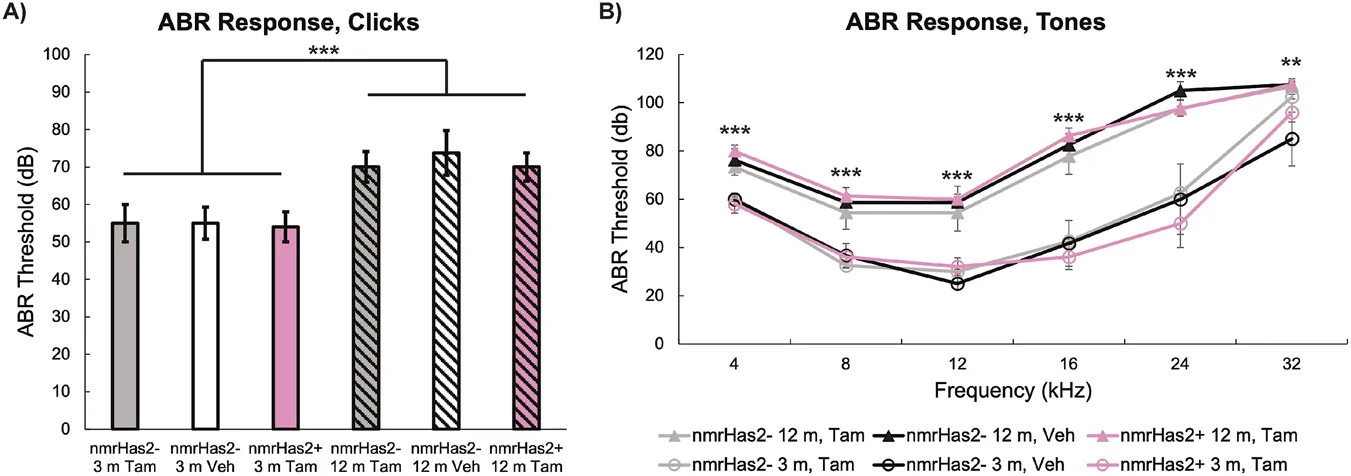
Results of auditory brainstem response (ABR) testing when 3- and 12-month-old mice that were stimulated with **(A)** clicks and **(B)** pure tones. Expression of naked mole-rat hyaluronan synthase 2 was induced in mice carrying the transgene (*nmrHas2+*) using intraperitoneal tamoxifen (Tam) injections at 1 month of age. Control mice without the transgene (*nmrHas2-*) were given either Tam or corn oil vehicle (Veh). The ABR thresholds (means ± SEM) were higher for clicks and tones in old mice compared to young mice, regardless of genotype or tamoxifen treatment, indicating *nmrHas2* did not attenuate age-related hearing loss. For differences based on age, ** indicates *P <* 0.01 and *** indicates *P* < 0.001. Sample sizes: *nmrHas2-* 3 m Tam (*n* = 4); *nmrHas2-* 3 m Veh (*n* = 6); *nmrHas2-* 12 m Tam (*n* = 9); *nmrHas2-* 12 m Veh (*n* = 8); *nmrHas2+* 3 m Tam (*n* = 5); *nmrHas2+* 12 m Tam (*n* = 8).

If AHL was attenuated by *nmrHas2* expression, this would manifest as decreased ABR thresholds. For clicks and all tones, ABR thresholds were significantly different based on age (*P* ≤ 0.004, Figure 2; Supplementary Table S1), but not based on genotype (*P* ≥ 0.37). Planned *post hoc* contrasts demonstrated the mean ABR thresholds were greater in old *nmrHas2-*mice for clicks and all tones compared to young *nmrHas2-*mice (*P* ≤ 0.006, Supplementary Table S1). The ABR threshold was greater in old *nmrHas2+* mice for clicks and tones at 4, 8, 12, 16, and 24 kHz compared to young *nmrHas2+* mice (*P* ≤ 0.001) but did not achieve statistical significance at 32 kHz (*P* = 0.08, Supplementary Table S1). The ABR threshold was also not statistically different between old *nmrHas2-* and *nmrHas2+* mice for clicks (*P* = 0.26, Figure 2A) and all tones (*P* ≥ 0.26, Figure 2B).

### 3.2 Cochlear expression of *nmrHas2*

We used extracted RNA to validate expression of *nmrHas2* in *nmrHas2+* mice. Zhang et al. (2023) established overall *Has2* tissue expression was substantially greater in *nmrHas2+* mice following Tam induction. We followed their qRT-PCR methods to evaluate cochlear *Has2* expression. Primers preferential for *nmrHas2* yielded a single-band product from NMR ovarian cDNA libraries while *musHas2* primers yielded no product from the same NMR libararies. Expression of *Has2* was substantially greater in the cochleae of *nmrHas2+* mice compared to nmrHas2- mice (Fold change = 781.98 ± 234.89 and 1.00 ± 0.38, respectively). These data indicated successful induction of expression of *nmrHas2* in the cochleae of *nmrHas2+* mice.

## 4 Discussion

The goal of this experiment was to determine whether ubiquitous expression of the *nmrHas2* transgene, including within the cochleae, attenuated AHL. Our results indicate that a genetic intervention that had previously been shown to improve healthspan, reduce inflammaging, and increase longevity in C57BL/6 mice (Zhang et al., 2023) did not attenuate AHL, i.e., these outcomes were decoupled in *nmrHas2* mice, which contrasts with outcomes following CR (Someya et al., 2007; Someya et al., 2010).

The early onset of AHL in C57BL/6 mice secondary to the variant SNP in *Cdh23* enabled us to add ABR testing to our larger study on the effects of the *nmrHas2* transgene on female reproductive aging at 12 months of age. It appears that, unlike CR, the anti-inflammatory properties of vHMM-HA observed in a previous study involving *nmrHas2* mice (Zhang et al., 2023) could not overcome the hair cell intrinsic hearing loss caused by the *Cdh23* variant. We elected to use C57BL/6 *nmrHas2* mice that carried the *Cdh23* variant, as did Zhang et al. (2023) for consistency, because this assured signs of AHL would be apparent at the end point of our larger study on reproductive aging in *nmrHas2* mice. One potential reason for the lack of an effect might be that while *nmrHas2* was expressed in the cochlea, it did not generate sufficient vHMM-HA to attenuate AHL. Alternatively, even if vHMM-HA was produced within the cochleae, it might have been markedly reduced owing to the high hyaluronidase activity in mouse tissues compared to the NMR (Tian et al., 2013).

If AHL had been attenuated in *nmrHas2* mice, our mechanistic hypotheses would have considered the means by which NMRs live exceptionally long and healthy lives. Whereas the specific cytoprotective mechanisms induced by vHMM-HA in the NMR are not fully understood, the cytoprotective pathway regulated by the transcription factor nuclear erythroid 2-related factor 2 (NRF2) is upregulated in the NMR relative to mice (Lewis et al., 2015). Typically, Kelch-like ECH-associated protein 1 (KEAP1) sequesters NRF2 in the cytoplasm and leads to NRF2 ubiquitination and degradation (Lewis et al., 2010). However, during periods of stress, a conformational change in KEAP1 allows NRF2 translocation to the nucleus to upregulate cytoprotective pathways (Suzuki and Yamamoto, 2017). In the NMR, KEAP1 is downregulated, and NRF2 and its downstream targets are continuously upregulated (Lewis et al., 2015). In a KEAP1 knock down mouse model, the NRF2-regulated pathways were continuously upregulated, and AHL in C57BL/6 mice was attenuated at 12 months of age (Oishi et al., 2020). Conversely, loss of NRF2 in knockout mice accelerated progression of AHL due to earlier loss of cochlear hair cells (Hoshino et al., 2011). As a caveat, the regulation of NRF2 in the cochlea from vHMM-HA expression remains to be determined. Additionally, vHMM-HA binds to the widely expressed CD44 receptor (Takasugi et al., 2020), and within the cochlear sensory epithelium, only outer pillar cells express CD44 (Hertzano et al., 2010). Previous findings indicate that CD44 is necessary for the anti-aging effects of vHMM-HA in NMRs (Takasugi et al., 2020); however, our data suggest cochlear hair cells in *nmrHas2* mice were not rescued by the changes in the extracellular environment, possibly because they do not express CD44. Collectively, the previous studies that proposed vHMM-HA and NRF2 as mechanistic explanations for of the NMR’s remarkable healthspan and longevity provided the impetus for us to hypothesize that these same mechanisms might apply to the *nmrHas2* mouse if AHL were mitigated in this model.

For future studies, a mouse strain other than C57BL/6 for which AHL occurs through a different mechanism could be informative. For example, AHL in CD/1 mice has been attributed to inflammation-induced aging of the cochleae (Riva et al., 2007). Therefore, CD1 mice might be useful for assessing the potential cytoprotective effects of *nmrHas2* expression in the cochlea. Because inflammation is a common sequela to noise or drug damage, it might be worth evaluating whether vHMM-HA can mitigate hearing loss in damage paradigms through its action on endogenous cochlear macrophages (Sato et al., 2010). Lastly, it would be interesting to determine whether vHMM-HA can alleviate the oxidative stress observed in the mouse cochlea from exogenous factors, such as cigarette smoke exposure (Paquette et al., 2018).

Overall, we did not observe an attenuation of AHL in mice that ubiquitously express *nmrHas2*. However, these data raise questions regarding the mechanism of the cytoprotective effect of vHMM-HA, as only outer pillar cells, and distinctly not outer hair cells, express CD44, a primary receptor for HA. Future studies examining cochlear cell-type specific HA-interactions and exposure to various exogenous factors that cause hearing loss could elucidate the primary cyptoprotective mechanism associated with *nmrHas2* expression.

## 5 Limitations

This research was limited to assessments of female mice because it was a supplemental study of the effects of ubiquitous *nmrHas2* transgene expression on female reproductive aging. The negative results did not warrant any further interrogation of cochleae other than to demonstrate that the transgene was expressed in this organ. However, the presence of the *nmrHas2* transgene in the cochleae does not ensure the presence of nmrHAS2 protein nor the presence of vHMM-HA.

## 6 Conclusion

Though CR mice and *nmrHas2* mice have been shown in previous studies to share phenotypes such as improved healthspans, reduced inflammaging, and longer lifespans, only CR alleviated AHL in C57BL/6 mice, whereas *nmrHas2* mice showed no attenuation of AHL. This points to differences in the mechanisms of action of these two interventions, at least within the cochleae.

## Data Availability

The raw data supporting the conclusions of this article will be made available by the authors without undue reservation.

## Supplementary material

SUPPLEMENTAL FIGURE 1 Products from qRT-PCR for hyaluronan synthase 2 (*Has2*) from the cochleae of 3-month-old mice that have transgenic expression of naked mole-rat (NMR) version of *Has2* (*nmrHas2+*) or control mice without transgenic expression of *nmrHas2* (*nmrHas2-*). cDNA derived from a NMR ovary was used as a positive control for *nmrHas2* and primers specific for mouse *Has2* as a positive control for the qRT-PCR. Primers for qRT-PCR were preferential for *nmrHas2* (Forward primer: GAG AGC TCA CAG GTG ACA CAG; Reverse primer: GGA GTC ACA AAC CTG CAC ATA ATC CAC A).
