# Supplementary material for: Age-related hearing loss persisted in mice that had demonstrated improved healthspan, reduced inflammaging and extended lifespan following expression of the naked mole-rat transgene for hyaluronan synthase 2
Source: Front Aging. 2026 Aug 27;7:1936677. doi: 10.3389/fragi.2026.1936677 (PMC13558403; doi:10.3389/fragi.2026.1936677)
Supplement: Supplementary file 1 [file Table1.docx]

**Supplementary Information**

| Table S1: Summary of auditory brainstem response (ABR) thresholds for clicks and pure tones in mice with and without naked mole-rat transgene hyaluronan synthase 2 (*nmrHas2*) at 3 and 12 months of age. Data is presented as least square means ± standard error of the mean. The *P-*value represents the value for each fixed effect in the generalized linear model. | | | | | | | | | |
| --- | --- | --- | --- | --- | --- | --- | --- | --- | --- |
|  |  | ABR Threshold (dB) | | | | | | | |
|  |  |  | Tones | | | | | | |
| **Age** | **Genotype × Treatment** | **Clicks** | | **4 kHz** | **8 kHz** | **12 kHz** | **16 kHz** | **24 kHz** | **32 kHz** |
| 3 months | nmrHas2- × Vehicle | 55 ± 4.3 | | 60 ± 2.6 | 37 ± 4.9 | 25 ± 2.2 | 42 ± 9.5 | 60 ± 14.6 | 85 ± 11.2 |
|  | nmrHas2- × Tamoxifen | 55 ± 5.0 | | 60 ± 10.8 | 33 ± 7.5 | 30 ± 7.1 | 43 ± 9.5 | 63 ± 14.4 | 103 ± 4.8 |
|  | nmrHas2+ × Tamoxifen | 54 ± 4.0 | | 58 ± 3.7 | 36 ± 2.4 | 32 ± 3.7 | 36 ± 5.1 | 50 ± 10.0 | 96 ± 4.0 |
| 12 months | nmrHas2- × Vehicle | 74 ± 6.0 | | 76 ± 6.3 | 59 ± 6.1 | 59 ± 6.7 | 83 ± 7.0 | 105 ± 3.8 | 108 ± 2.5 |
|  | nmrHas2- × Tamoxifen | 70 ± 4.1 | | 73 ± 2.4 | 54 ± 6.9 | 54 ± 7.7 | 78 ± 7.4 | 98 ± 3.2 | 107 ± 2.4 |
|  | nmrHas2+ × Tamoxifen | 70 ± 3.8 | | 80 ± 1.9 | 61 ± 4.0 | 60 ± 3.8 | 86 ± 3.2 | 98 ± 1.6 | 108 ± 1.6 |
| *P-*value | Age | <0.001 | | <0.001 | <0.001 | <0.001 | <0.001 | <0.001 | 0.004 |
|  | Genotype | 0.983 | | 0.547 | 0.367 | 0.426 | 0.819 | 0.460 | 0.856 |
|  | Treatment | 0.589 | | 0.658 | 0.438 | 0.869 | 0.682 | 0.593 | 0.737 |
|  | Age × Genotype | 0.892 | | 0.352 | 0.742 | 0.877 | 0.320 | 0.583 | 0.218 |
